# Endoscopically assisted reconstruction of chronic Achilles tendon ruptures and re-ruptures using a semitendinosus autograft is a viable alternative to pre-existing techniques

**DOI:** 10.1007/s00167-022-06943-2

**Published:** 2022-04-09

**Authors:** Niklas Nilsson, Baldvin Gunnarsson, Michael R. Carmont, Annelie Brorsson, Jón Karlsson, Katarina Nilsson Helander

**Affiliations:** 1 Department of Orthopaedics, Institute of Clinical Sciences, Sahlgrenska Academy, University of Gothenburg, Göteborgsvägen 31, Mölndal, 431 80 Gothenburg, Sweden; 2 Department of Orthopaedics, Sahlgrenska University Hospital, Mölndal, Sweden; 3 IFK Kliniken Rehab, Gothenburg, Sweden; 4 Department of Orthopaedic Surgery, Princess Royal Hospital, Shrewsbury and Telford Hospital NHS Trust, Shropshire, UK

**Keywords:** Chronic Achilles tendon rupture, Semitendinosus graft, Achilles tendon re-rupture, Surgical repair, Endoscopically assisted technique, ATRS

## Abstract

**Purpose:**

Achilles tendon ruptures are termed chronic after a delay in treatment for more than 4 weeks. The literature advocates surgical treatment with reconstruction to regain ankle push-off strength. The preferred technique is, however, still unknown and is often individualized. This study aims to present the technique and clinical outcome of an endoscopically assisted free semitendinosus reconstruction of chronic Achilles tendon rupture and Achilles tendon re-ruptures with delayed representation. It is hypothesized that the presented technique is a viable and safe alternative for distal Achilles tendon ruptures and ruptures with large tendon gaps.

**Method:**

Twenty-two patients (13 males and 9 females) with a median (range) age of 64 (34–73) treated surgically with endoscopically assisted Achilles tendon reconstruction using a semitendinosus autograft were included. The patients were evaluated at 12 months post-operatively for Achilles tendon Total Rupture Score (ATRS), calf circumference, Achilles Tendon Resting Angle (ATRA), heel-rise height and repetitions together with tendon length determined by ultrasonography, concentric heel-rise power and heel-rise work.

**Results:**

The patients reported a median (range) ATRS of 76 (45–99) out of 100. The median (range) ATRA on the injured side was 60° (49°-75°) compared with 49.5° (40–61°), *p *< 0.001, on the non-injured side. Eighteen out of 22 patients were able to perform a single-leg heel-rise on the non-injured side. Sixteen patients out of those 18 (89%) were also able to perform a single heel-rise on the injured side. They did, however, perform significantly lower number of repetitions compared with the non-injured side with a median (range) heel-rise repetitions of 11 (2–22) compared with 26 (2–27), (*p* < 0.001), and a median (range) heel-rise height of 5.5 cm (1.0–11.0 cm) compared with 9.0 cm (5.0–11.5 cm), (*p* < 0.001). The median calf circumference was 1.5 cm smaller on the injured side, 37.5 cm compared with 39 cm, when medians were compared. The median (range) tendon length of the injured side was 24.8 cm (20–28.2 cm) compared with 22 cm (18.4–24.2 cm), (*p* < 0.001), on the non-injured side.

**Conclusion:**

The study shows that endoscopically assisted reconstruction using a semitendinosus graft to treat chronic Achilles tendon ruptures and re-ruptures with delayed representation produces a satisfactory outcome. The technique can restore heel-rise height in patients with more distal ruptures or large tendon defects and is therefore a viable technique for Achilles tendon reconstruction.

**Level of evidence:**

IV.

## Introduction

Several methods of repair and reconstruction have been presented to restore heel-rise height and push-off strength in patients with chronic ruptures and re-ruptures with delayed representation. In many cases, direct end-to-end repair may not be possible due to large separation of the tendon ends and the formation of intermediate scar tissue. Reconstruction is required when there is a defect between the tendon ends [28]. Various surgical techniques include the augmentations of end-to-end repairs, interposition grafts and tendon transfers. There are few comparative studies between reconstructive techniques. None of the reported techniques have been shown to produce superior outcome [23]. It is generally recommended to use V–Y tendon transfer [12, 20] or a gastrocnemius aponeurosis flap [8] in tendon ruptures with a gap that is less than 5 cm. In tendon ruptures with larger defects (> 5 cm) the preferred method is using either flexor hallucis longus (FHL) grafts [32, 33] or semitendinosus grafts [22, 36].

Semitendinosus tendon graft is routinely used in knee surgery for ligament reconstructions and it has also been shown to have good outcomes when used to treat chronic ATR [22, 35, 40]. Most of these studies have involved open reconstructions performed through a large single incision, with potential wound healing complications as reported for repairs of acute ruptures [10, 11].

This study reports a new surgical technique that involves an endoscopically assisted reconstruction of the Achilles tendon, using ipsilateral semitendinosus autograft. A similar method has previously been described by Li et al. although the outcomes reported did not include validated measures specific for the function of the Achilles tendon [19]. The purpose of this study is to present and evaluate the one-year results and safety of the reported technique, using validated outcome measures. It was hypothesized that this is a viable technique to treat patients with large tendon defects, distal ruptures and patients with an increased risk of post-operative wound infections.

## Method and materials

Twenty-two patients operatively treated using a semitendinosus autograft between 2016 and 2020 were included in the study. All patients had a clinically diagnosed chronic ATR (20 patients) or a re-rupture with delayed representation (2 patients). The inclusion criteria were any unilateral Achilles tendon rupture that could not be surgically reconstructed using direct repair or reinforced using a gastrocnemius aponeurosis flap. The reason was either a large tendon gap (> 5 cm), distal rupture or previous reconstruction to the Achilles tendon. The exclusion criteria were low functional demands or comorbidities that excluded surgery as a treatment option.

Eight patients (5 women and 3 men) were treated at the Sahlgrenska University Hospital in Mölndal, Sweden, and 14 patients (4 women and 10 men) at the Princess Royal Hospital, Shrewsbury and Telford Hospital NHS Trust, Shropshire, United Kingdom. The median age was 64 years (range 34–73). Median time from injury to surgery was 5 months (range 1.5–139). The tendon gap was either measured using ultrasonography (*n* = 10) or MRI (*n* = 7) prior to surgery. The median tendon gap (*n* = 17) was 5 cm (2–10 cm). The patient demographics are presented in Table 1. Ethical approval was obtained from the ethical review authority in Sweden (DNR 2020-02971) and all patients in Sweden signed an informed consent before inclusion. The British patients were included as part of standardized clinical service evaluation. All surgeries were performed by the same two surgeons (MC/KNH).

**Table 1:** Demographics for included patients including nationality, tendon gap and treatment delay in months

Patient demographics (*n* = 22)	Mean (SD) median (range)
Age, y	60 (12)
	64 (34; 73)
Patient sex	
Male	13 (59%)
Female	9 (41%)
Patient nationality	
Sweden	8 (36.4%)
United Kingdom	14 (63.6%)
Tendon gap (cm) (*n* = 17)	5.24 (2.16)
	5 (2; 10)
Delay in treatment (months)	11.1 (28.7)
	5.0 (1.5; 139)

### Surgical technique

The surgery was performed under spinal or general anaesthesia using a tourniquet. The patient was placed in a semi-prone position to allow easier access to both the semitendinosus graft and the ruptured Achilles tendon [5]. With the knee flexed to 90˚ a 2-cm longitudinal incision was made over the pes anserinus and the semitendinosus tendon was identified and harvested. The graft was later prepared with Nr. 2 Orthocord (Depuy Synthes, Johnson and Johnson) whip-stitch sutures at both ends. The graft was then wrapped in a gauze soaked in vancomycin solution. The sartorius fascia was closed using a Nr. 0 polyglactin suture (Vicryl, Ethicon, Johnson and Johnson) and the skin incision was closed using non-absorbable sutures.

**Fig. 1:**
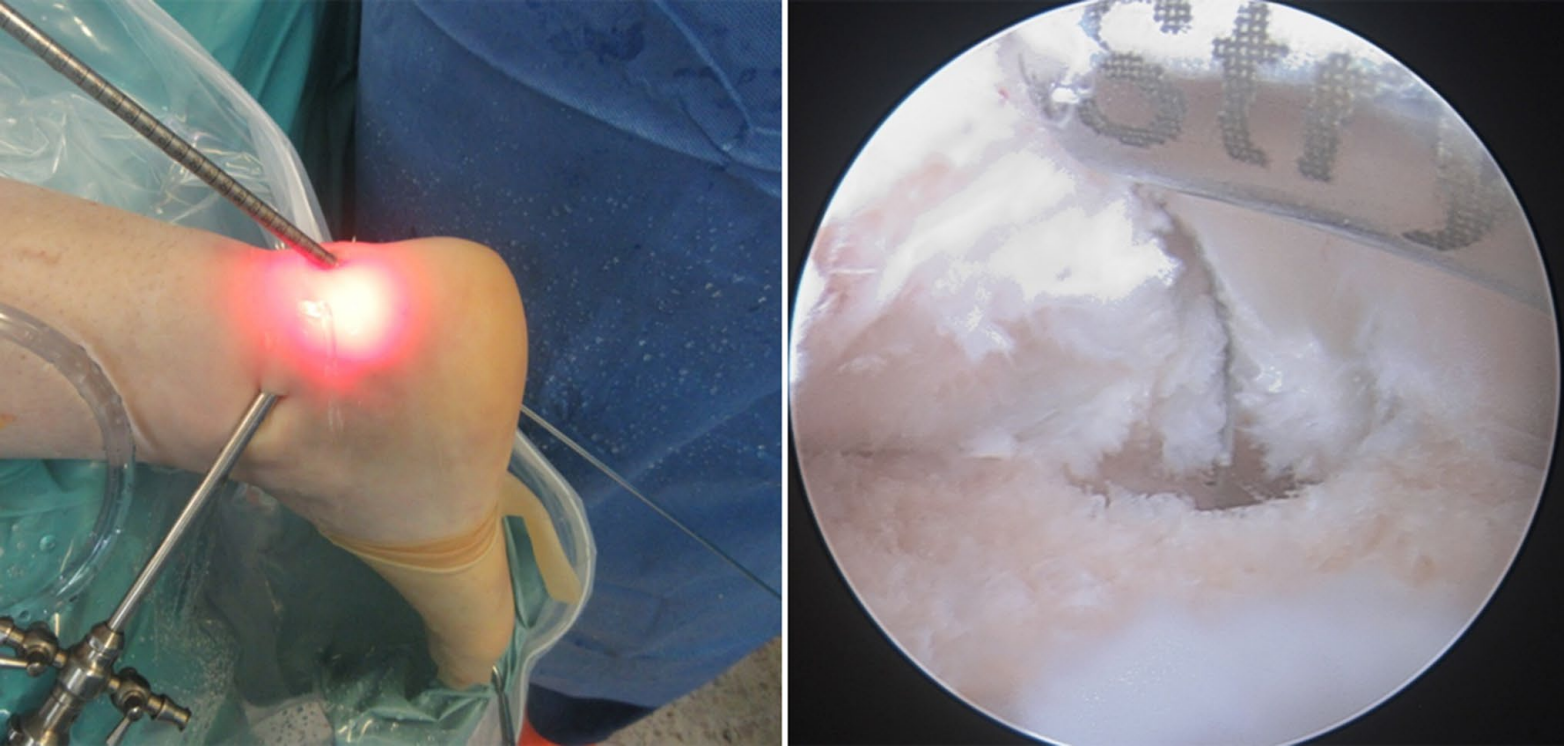
An endoscopic calcaneoplasty using standard posteromedial, posterolateral and accessory posterolateral portals was performed using a bone shaving burr at the level of the Achilles tendon insertion. A guide-wire was positioned and passed through the calcaneum bone under endoscopic visualization

Standard posteromedial, posterolateral and accessory posterolateral portals were created (Fig. 1) [6, 44]. An endoscopic calcaneoplasty was performed using a bone shaving burr to the level of the Achilles insertion to allow full visualization of the future tunnel site. Under direct and endoscopic vision, a midline tenotomy was performed onto the calcaneum and a guide-wire was positioned on the deep side of the tendon. The guide-wire was then passed through the calcaneum towards the insertion of the plantar fascia and a 7-mm tunnel was drilled through the calcaneus (Fig. 1).

**Fig. 2:**
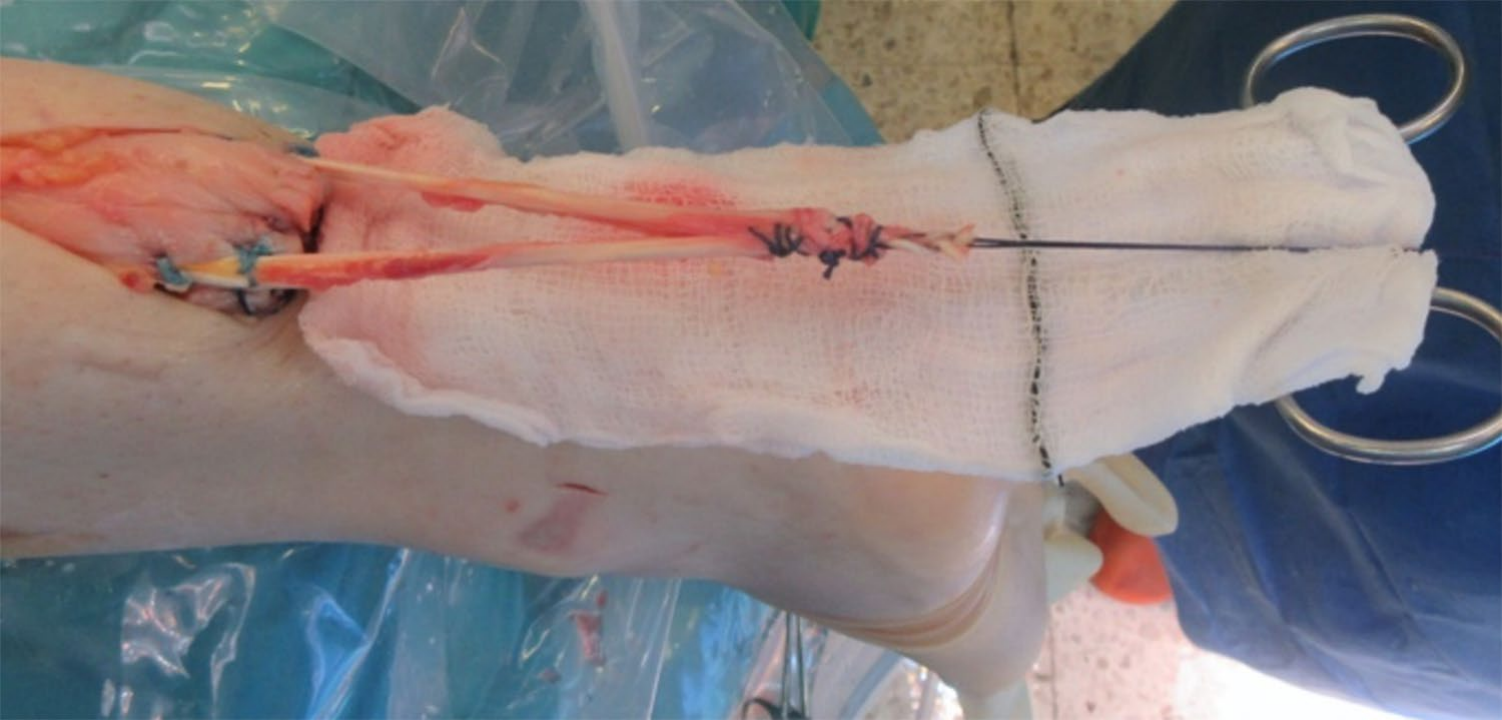
The proximal tendon end was exposed using a 4–5 cm longitudinal incision. The standardized semitendinosus graft was passed through a coronal tenotomy to produce equal lengths on the lateral and medial side. The proximal tendon end was reinforced using running locking sutures

The proximal end of the tendon was palpated at the level of the distal calf. A 4-5 cm longitudinal incision was made over the proximal tendon end and released from the scar tissue. Care was taken to minimize skin contact of the graft using a sterile gauze. The transected tendon end was then reinforced using a running locking Nr. 2 polyglactin suture (Vicryl, Ethicon, Johnson and Johnson). At 2 cm proximal to the tendon end, a coronal tenotomy was performed using an 11 scalpel and the graft was then passed through the tenotomy to produce equal medial and lateral lengths (Fig. 2). The graft was secured at the tenotomy and the distal end of the tendon with Nr. 2 Orthocord locking sutures (Depuy Synthes, Johnson and Johnson).

**Fig. 3:**
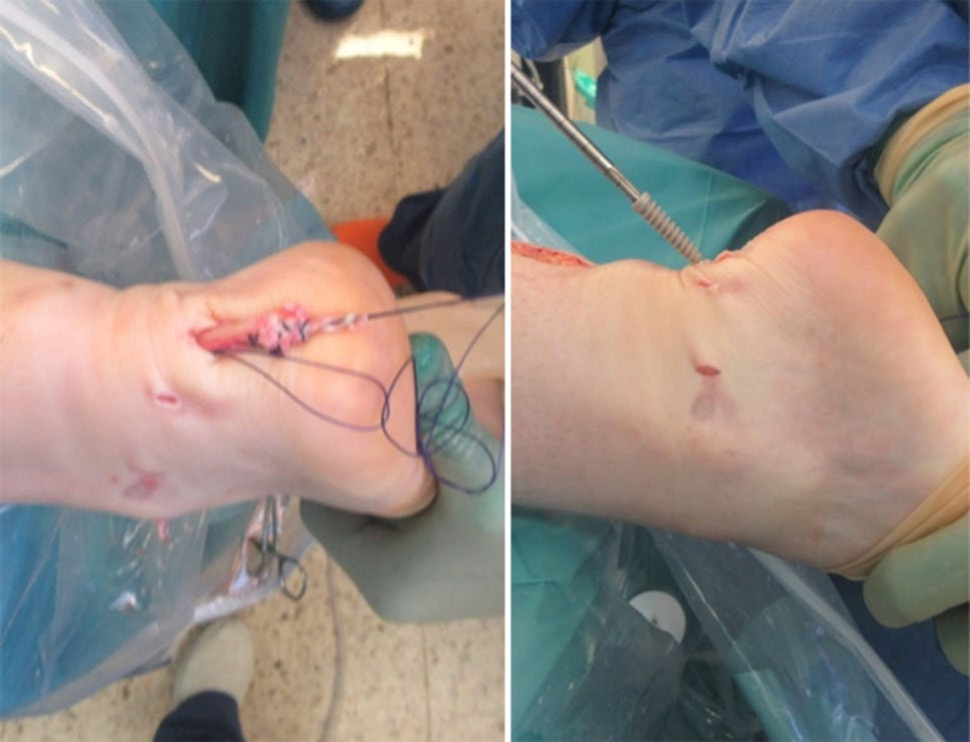
The semitendinosus graft was threaded down towards the distal tendon end and then through the calcaneal tunnel. The semitendinosus graft was held in maximal tension and then secured using a 7–8 mm interference screw from the posteromedial incision

**Fig. 4:**
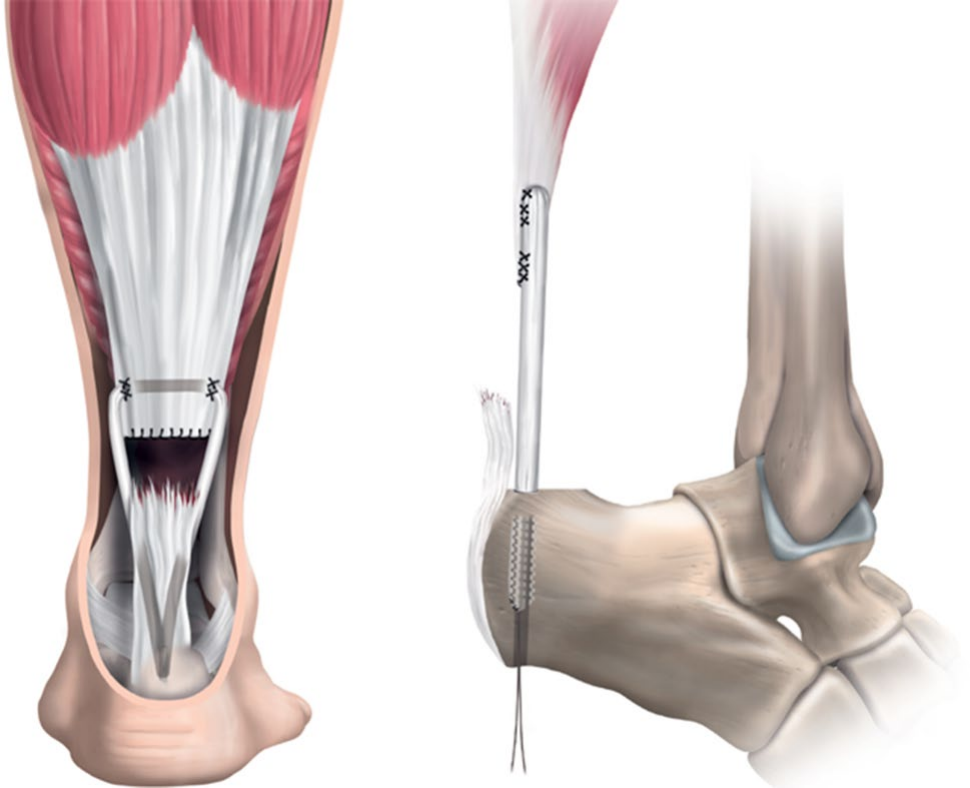
Post-operative anatomical pictures describing the graft positioning and channelling as well as the location of the fixation screw in the calcaneum

Using forceps, a subcutaneous passage for the graft to the midline tunnel incision was performed and the graft passed (Fig. 3). The graft was then threaded through the calcaneal tunnel and drawn out through the plantar exit wound. The ankle was held in full plantar flexion and the graft was pulled through the calcaneal tunnel and held in maximal tension. The graft was secured with a 7–8 mm interference screw, which was placed through the midline incision and into the calcaneus (Fig. 3). All wounds were closed using non-absorbable sutures, dressings and padding together with a full below-the-knee cast applied in an equinus position (Fig. 4).

### Post-operative immobilization and medication

The leg was immobilized in a circumferential synthetic cast with ankle in an equinus position. Patients were mobilized non-weight-bearing using two crutches. At 3 weeks following surgery sutures were removed and the cast was changed to a Walker-boot with 3–4 heel wedges and an anterior plaster splint to prevent dorsiflexion of the ankle. Partial weight-bearing was permitted in the Walker-boot from 3 weeks and full weight-bearing was commenced at 6 weeks. The Walker-boot was removed at 8 weeks post-operatively. Thromboprophylaxis with Fragmin 5000 IE daily was used for 2–3 weeks.

### Post-operative evaluation

The surgical technique and post-operative immobilization were similar for the designated hospitals. There were, however, minor differences in terms of the follow-up. All patients were evaluated using a validated functional test battery with additional clinical measurements and patient-reported outcomes at 12 months post-operatively. This evaluation included the Achilles tendon Total Rupture Score (ATRS), the Achilles Tendon Resting Angle (ATRA), the calf circumference, the heel-rise height measured with a tape from floor to the patients heel and the heel-rise repetitions at 12 months [38]. The ATRS was used to evaluate the patients’ self-perceived Achilles tendon function [30]. Tendon length was assessed using ATRA [4]. At the Sahlgrenska University Hospital additional measurements using a linear encoder with software (Ergotest, MuscleLab, Oslo, Norway) were performed to evaluate power and muscle endurance in terms of concentric power (watts) and work capacity (joules). At the Sahlgrenska University Hospital ultrasonography [39] was also implemented to further estimate the tendon length. For comparison of patients, the limb symmetry index (LSI) was used in all functional tests and clinical measurements [38]. All evaluations were performed by two experienced evaluators of Achilles tendon injuries.

### Concentric heel-rise power (watts)

The concentric power test was performed with the patient performing a single-leg heel rise as powerful and quickly as possible in a weight machine with or without added weights. At first, 13 kg was added, the weight was then increased with 10 kg for each interval until a decrease in power output was noted. Each interval was 3 repetitions with 15-s rest between each repetition. The highest force in watts was determined relative to the patient’s weight [37].

### Heel-rise test: work capacity (joules)

The heel-rise test for work capacity was performed with the patient performing single-leg heel-rises on a box with 10° incline at a rate of 30 repetitions/minute. The test was ended when the patient was unable to continue, or when the heel rises were lower than 2 cm. Software from MuscleLab was used to calculate total work by measuring total height of heel-rises × body weight. Number of repetitions and height of each repetition was also registered [38].

### Statistical analysis

Statistical calculations were performed using IBM SPSS Statistics, Version 28, IOS, Mac. Descriptive data were reported as the mean (standard deviation, SD) and the median (range). The Limb Symmetry Index (LSI) was defined as the ratio between the injured side and the healthy side (injured/healthy × 100 = LSI). Non-parametric tests were used for analysis of outcomes. Wilcoxon’s signed-rank test was used for comparison between the injured and the non-injured side. *P* values < 0.05 were considered statistically significant. No sample size or power calculation was performed as all available patients were included and no adequate control group could be identified.

## Results

At the 12-month follow-up the patients reported a median ATRS score of 76 (45–99). Eighteen out of 22 patients were able to perform a single-leg heel-rise on the non-injured side. Sixteen out of those 18 (89%) were also able to perform heel-rises on the injured side. However, the median height and number of repetitions were significantly lower on the reconstructed side as shown in Table 2. The tendon length was longer by a mean of 2.8 cm on the injured side compared to the non-injured side.

**Table 2:** Post-operative clinical measurements regarding ATRS, ATRA, heel-rise height and reps, calf circumference and tendon length at 12 months. Tendon length measurements using ultrasound were only measured in patients evaluated in Sweden

	Injured side	Non-injured side	LSI %	Difference injured vs non-injured side
	Median (IQR) min–max	Median (IQR) min–max	Median min–max	*p* value
ATRS (*n* = 22)	76 (35.5)	**–**	**–**	–
	45; 99			
ATRA (degrees) (*n *= 20)	60 (15)	49.5 (6)	83	< 0.001
	49; 75	40; 61	69; 96	
Heel-rise height (cm) (*n* = 16)	5.5 (5.75)	9.0 (2.75)	61	< 0.001
	1.0; 11.0	5.0; 11.5	20; 100	
Heel-rise reps (*n* = 11)	11 (18)	26 (14)	42.3	< 0.001
	2; 22	2; 30	7.6; 81.5	
Calf circumference (cm) (*n* = 20)	37.5 (6)	39 (6.4)	96	< 0.001
	30; 46	30.0; 50.0	87; 100	
Tendon length (cm) (*n* = 6)	24.8 (6)	22 (5.2)	91	< 0.001
	20; 28.2	18.4; 24.2	86; 92	

The concentric power test and heel-rise work test were only performed at the Sahlgrenska University Hospital. Seven out of those eight patients were able to complete the concentric power test with a median LSI of 70% (0–103.3). Seven patients were able to complete the heel-rise work test with a median LSI total work of 3.9% (0–60.8) and a median LSI of repetitions of 8% (0–81.5). The median LSI heel-rise height was 30% (0–79). These results are presented in Table 3.

**Table 3:** Functional tests using MuscleLab®

	Injured side	Non-injured side	LSI %	Difference between injured and non-injured side
	Median (IQR) min;max	Median (IQR) min;max	Median min;max	*p* value
Concentric power (W) (*n* = 7)	7 (223)	224 (281)	70	0.004
	0; 317	10; 945	0; 99	
Heel-rise total work (J) (*n* = 7)	65 (412)	1325 (1220)	3.9	< 0.001
	0; 962	227; 1650	0; 60.8	

Of the 22 patients in the present study, only two had minor post-operative wound complications. The surgical wounds had not fully healed at the first clinical control 2 weeks post-operatively. The patients were treated with oral antibiotics for 7 days and the wounds healed uneventfully. Another patient had an iatrogenic sural nerve injury with sensory loss on the lateral side of the foot. There were no re-ruptures within the patient group.

## Discussion

The most important finding of this study is that the endoscopically assisted reconstruction of the Achilles tendon using an ipsilateral semitendinosus autograft is a valuable technique that can successfully be used to treat chronic ATR and re-rupture with delayed representation. The technique described in this report produced a satisfactory outcome in terms of patient-reported outcome measures and clinical evaluation. By endoscopically assisting the reconstruction, it was possible to minimize the size of the surgical incision. The repair of a chronic ATR has previously been known to have a higher rate of wound complications due to the need of a more extensive surgical approach [7].

In a previous study by Nilsson-Helander et al. [29], an open reconstruction using a gastrocnemius flap was performed in 28 patients affected by chronic ATR or re-rupture. The patients reported a median ATRS of 83 at 2 years following reconstruction. In the present study, patients reported an ATRS of 74 at the 12-month follow-up. Nilsson-Helander et al. [29] did consider their technique to be proficient in tendon gaps up to 6 cm. In the present study, the semitendinosus graft has been shown to be efficient in reconstructing tendon gaps extending to 10 cm. Lever et al. [18] presented a technique using flexor hallucis longus graft to treat patients with larger tendon gaps, more comparable to this study, with a mean post-operative ATRS of 83 at a mean follow-up time of 73 months (29–120). However, the ATRS and functional outcomes are likely to be higher after a prolonged period of rehabilitation as shown for acute ruptures [31]. Due to the different inclusion criteria, diverse study designs and heterogeneous patient characteristics, direct comparisons between studies on chronic ATR are difficult to make.

Systematic reviews of surgical technique for acute ATR have showed a lower risk of post-operative complications for minimally invasive technique compared with open repair [11]. Gatz et al. [10] reported lower risk of wound complications with minimally invasive techniques, however, nerve injury was more frequent. It is likely that these complications may be related to greater incision length and surgical dissection. It must be appreciated, however, that a small incision which is stretched during surgery may be more prone to wound breakdown due to vascular compression and bruising of the wound edges. To the best of our knowledge no direct comparison of open versus minimally invasive techniques have been reported for chronic ATR or re-ruptures.

A variety of other tendon grafts have been described for repair of chronic ATR and re-ruptures [1, 3, 9, 15–17, 21, 42, 45]. Many of them involve local tendon transfer to the injured Achilles tendon, for example the flexor hallucis longus and peroneus brevis [27, 32]. The transfer of these local grafts has the potential to weaken the foot, reduce stability and in turn affect a patient’s balance and gait [2, 13, 34]. The use of the semitendinosus, therefore has the advantage that it does not directly alter foot function and that it has also been reported to regenerate [41]. The advantage of calcaneal tunnel fixation is that a distal Achilles stump is not required for reattachment [24].

Open and minimally invasive techniques using a semitendinosus graft, both allograft and autograft, have been described [14, 22, 25, 36, 40, 43]. Those studies report ATRS scores between 86 and99. In the present study, there is a higher mean age and a shorter follow-up time of 12 months, compared with commonly over 30 months in other studies. It does, however, show that patients improve in heel-rise height and present satisfactory ATRS scores within one-year post-operatively. Studies using other techniques for repair of chronic ATR, aponeurosis flap [29], V–Y plasty operation [20], flexor hallucis longus transfer, peroneus brevis transfer [26] or direct repair [46], have reported an ATRS of 83–94 points. Instead, these studies included younger groups of patients with a mean age 46, 38.5, 39, and 52.7 years, respectively, and a minimum follow-up of 2 years. Olsson et al. [31] showed in a study from 2011 that for acute ATR, the ATRS increased in most patients. Moreover, the ATRS score increased significantly in the non-operatively managed group between the 12 months’ follow-up and 24 months. Given that patients with chronic Achilles tendon ruptures have muscle hypotrophy due to disuse and lack of muscular activity, prolonged rehabilitation should be employed until optimal functional outcome is attained following surgery.

Three patients were not able to perform the single-leg heel rise at the 12-month follow-up. However, one of the patients (age 72) reported an ATRS of 99 and was able to walk without any difficulties. It is therefore important to keep in mind the demands of the patients when selecting appropriate treatment and techniques.

The limitations of this report are firstly the limited number of patients included. Secondly, the ATRS was not assessed preoperatively, nor were the heel-rise height, concentric power and endurance test. The follow-up time was also only 12 months and it is therefore difficult to compare to previous results. Another drawback is that no standardized method of tendon gap measurement was used. A strength of this report is the usage of the validated outcome measurements, ATRS, the heel-rise tests and ATRA. A larger cohort size with a control group and a longer follow-up time are needed to give a more conclusive result on the efficacy of this technique compared with others. At this time no adequate control group could be identified, as previous methods applied were not regarded sufficient in patients with large tendon gaps.

## Conclusion

The study shows that endoscopically assisted reconstruction with a semitendinosus graft of chronic ATR and re-rupture with delayed representation produces a satisfactory outcome and can restore heel-rise height and is therefore a viable technique for Achilles tendon reconstruction. This minimally invasive technique may also be more applicable to patients with a pre-existing increased risk of wound complications, patients with a more distal rupture or patients with a tendon gap exceeding 5 cm.
